# Generation and identification of kokumi compounds and their validation by taste-receptor assay: An example with dry-cured lamb meat

**DOI:** 10.1016/j.fochx.2022.100218

**Published:** 2022-01-19

**Authors:** Jihan Kim, Raise Ahmad, Santanu Deb-Choudhury, Arvind Subbaraj, Julie E. Dalziel, Scott O. Knowles

**Affiliations:** Smart Foods Innovation Centre of Excellence, AgResearch Ltd., Te Ohu Rangahau Kai, Palmerston North 4422, New Zealand

**Keywords:** Taste, Hydrolysate, Meat crust, Kokumi, CaSR, γ-Glutamyl peptides

## Abstract

Kokumi tastants are small γ-glutamyl peptides (GGP) that enhance flavour in foods. We sought to generate GGP from the meat crusts of dry-cured lamb, an underutilised protein resource, identify these using mass spectrometry, and validate their functional activity using a kokumi-calcium sensing receptor (CaSR) assay. The water-soluble extract (WSE) of meat crust was hydrolysed by protease A (PA) and treated with glutaminase (GA). Fifteen GGP were identified, with 14 being significantly increased in PA and GA groups compared to WSE, as were along with free amino acid levels. The GA extract activated CaSR with higher potency and efficacy than PA and WSE suggesting the generation of potent kokumi tastants. The *in vitro* receptor assay might be an expedient tool for screening kokumi tastants prior to conducting human sensory analysis. Collectively, our findings indicate that the meat crust can be a valuable source to generate kokumi tastants via a two-step enzymatic reaction.

## Highlights

• Fifteen ɤ-glutamyl dipeptides (GGP) were identified in meat crust hydrolysate.
• *In vitro* cell based CaSR assay was applied to measure kokumi tastants functionality.
• Enriched extract of GGP following glutaminase treatment activated CaSR robustly.
• CaSR assay was used as an effective tool to screen kokumi tastants abundance in foods.
• Meat crusts can be a good source to generate kokumi tastants via enzymatic reactions.

## 1 Introduction

The surface that develops on aged and/or cured meat is called a 'crust'. The crust is typically dry, tough and unsightly and therefore trimmed and discarded, which can result in substantial loss of material and value. Up to 34% of a dry-aged beef striploin goes to waste (Degeer, Hunt, Bratcher, Crozier-Dodson, Johnson, & Stika, 2009). Recently, the meat crust has received attention as an underutilised resource of protein and peptides (Gallego, Toldrá, & Mora, 2022). Its addition to beef patties can enhance flavour, juiciness and oxidative stability (Park et al., 2018, Xue et al., 2021). This suggests that the meat crust has potential as a value-added ingredient.

Kokumi tastants enhance flavour and contribute to the rich taste of meaty foods. These compounds are found in nature (legumes, garlics, mushrooms) and they are abundant in fermented foods (cheese, soy sauce) as diverse γ-glutamyl peptides (GGP) (Yamamoto, 2019). GGP and other kokumi tastants bind to the calcium sensing receptor (CaSR) to transmit sensory signal transduction of kokumi taste perception (Ahmad & Dalziel, 2020). The kokumi flavour imparts a kind of non-physical thickness, mouthfullness and complexity to taste perception (Miyaki, Kawasaki, Kuroda, Miyamura, & Kouda, 2015). Notably, CaSR activation was positively correlated with kokumi taste attributes in human sensory studies suggesting receptor assay as a predictive indicator of enriched kokumi tastants in foods (Kim et al., 2021, Ohsu et al., 2010).

In order to increase the amount and availability of GGP in foods, studies have used combinations of protein hydrolysis and addition of glutaminase for the interconversion of Gln and Glu and for its γ-glutamyl transferase activity to transfer the γ-glutamyl moiety to other amino acids and peptides (Li et al., 2020, Sari et al., 2014). We suspect that they are particularly abundant in the crust. Thus, we hypothesize that although natural aging and ripening of cured meats generates kokumi tastants (or their precursors) in the crusts, their abundance can be increased by enzyme catalysis when the additional protease and glutaminase SD-C100S are added.

Although CaSR responds to kokumi taste substances, the application and efficiency of cell based CaSR assay in food industry has not been documented yet. This is the first study demonstrating potential of CaSR assay to screen an increment of kokumi tastants in food formulations prior to conducting sensory analysis. In the present study we extracted protein from meat crusts and identified the γ-glutamyl di-peptides using mass spectrometry. To increase kokumi taste substances, the meat crust was further subjected to protease hydrolysis and glutaminase reaction. Subsequently, fractions rich in GGP were tested for their ability to activate kokumi receptor in an *in vitro* cell-based assay.

## 2 Methods

### 2.1 Sample preparation

Dry-cured lamb legs were prepared in the style of prosciutto ham by a local charcuterie butchery. The fresh meat was trimmed of excess fat and connective tissues and then scrubbed with 2% salt (w/w) and subsequently hung in an air-circulated room (17 °C, 70% relative humidity) to dry for 21 days. As soon as the drying process was finished, the conditions was set up to 17 °C with 85% relative humidity for a ripening process (7 days). The final pH and water activity of dry-cured meats were 5.62–5.68 and 0.78–0.79 respectively. The samples after ripening process were immediately vacuum-packaged and stored at 4 °C until further analysis.

Approximately 50 g of meat crust was collected from each of three randomly selected legs (n = 3). The crusts were separately homogenised with 150 mL of Milli Q water for 1 min at room temperature. 50 g of each homogenate was stored at −80 °C until further analysis. The homogenate represents a water-soluble extract of untreated crust and was designated as ‘WSE’.

### 2.2 Enzymatic hydrolysis and γ-glutamylation

Protease A and Glutaminase SD-C100S were procured from Amano Enzyme Inc (Nagoya, Japan) and used according to the manufacturer’s instructions. The pH of an aliquot of crust homogenate WSE was adjusted to 7.0 and incubated at 50 °C for 10 mins in a water bath. Protease A was added at 0.5% (w/w) and the bath maintained at 50 °C for a further 5 hr with continuous shaking. At the end of the incubation period, the hydrolysate was heated at 90 °C for 15 mins to inactivate the enzyme. After cooling at room temperature, the pH of the homogenate was again adjusted to 7.0 then stored at −80 °C. The hydrolysate was designated as ‘PA’. The pH of an aliquot of PA was adjusted to 10.0 with 2 M NaOH, then Glutaminase SD-C100S was added at a ratio of 0.2 U/mL (1,132 U/g). After incubation at 37 °C for 5 hr, the homogenate was heated at 90 °C for 15 mins to inactivate the enzyme and then stored at −80 °C. The γ-glutamylated hydrolysate was designated as ‘GA’.

### 2.3 Quantitative analysis of free amino acids

For the WSE group, 1 g (wet basis) was mixed with 0.2 mL internal standard and 8.8 mL 0.1 M HCl. For the PA and GA groups, 40 µL was mixed with 20 µL internal standard and 940 µL 0.1 M HCl. In all cases a 500 µL aliquot was passed through a < 10 kDa molecular weight cut off (MWCO) filter, then 50 µL of filtrate was reacted with phenyl isothiocyanate. The derivatised free amino acids (FAA) were analysed by HPLC (LC-10AD VP, Shimadzu, Japan) equipped with a PicoTag® for free amino acid analysis column (3.9 × 300 mm, Waters Corporation, MA USA) according to the method described by White, Hart, and Fry (1986). Analytical batches consisted of a derivatization blank, a mix of proteinogenic amino acid standards (Sigma AAS18, A0884, G3126), and a composite of physiological amino acid standards (Sigma A6407, A6282). Results were calculated as absolute concentrations in the extracts.

### 2.4 Relative quantitative analysis of γ-glutamyl dipeptides

#### 2.4.1 Dipeptide extraction and chromatography

WSE, PA and GA were weighed (540 ± 21 mg) in 2 mL microcentrifuge tubes. A ceramic bead was added along with 1 mL of extraction solvent methanol:water (1:1, v/v). Samples were disrupted in a bead-mill homogeniser (TissueLyser II; Qiagen, CA USA) for 5 mins, rested at −20 °C for 1 hr, and then centrifuged for 2 × 10 mins (12,000 RPM, 4 °C). The supernatant (200 µL) was transferred to another microcentrifuge tube and evaporated to dryness in a SpeedDry rotational vacuum concentrator (Martin Christ RVC 2–18CD plus, Osterode, Germany) equipped with a PC 3001 pump and controller (Vacuubrand, Wertheim, Germany). Dried extracts were reconstituted in 200 µL of acetonitrile:water (1:1, v/v), centrifuged again for 10 mins (12,000 RPM, 4 °C), and 90 µL of the supernatant was transferred to a glass insert fitted in a 2 mL sampling vial. To this extract was added 10 µL of l-Tyrosine-3,3-d_2_ (10 μg/mL) as internal standard.

Peptides in the extracts were separated by ultra-high-performance liquid chromatography (UHPLC) (Nexera X2, Shimadzu, Japan) coupled to a LCMS-9030 quadrupole time-of-flight (Q-TOF) mass spectrometer equipped with an electrospray ionization source (Shimadzu, Japan). Sample (10 µL) was injected into a normal phase Ascentis® Express HILIC UHPLC column (2.1 × 100 mm, 2 µm particle size; Sigma, USA) and eluted at 30 °C over a 20-minute gradient with a flow rate of 400 µL/minute. The mobile phase solvent A was 10 mM ammonium formate in water and solvent B was acetonitrile with 0.1% formic acid. The solvent gradient program started at 97% solvent B from 0 to 0.5 mins, decreased to 70% within 11.5 mins and further to 10% from 11.5 to 13.5 mins, held at 10% for 1.5 mins, increased to 97% B within 1 min and held at that concentration until the end of the elution run.

#### 2.4.2 Mass spectrometry

Full scan (*m*/*z* 55–1100) and multiple reaction monitoring (MRM) windows were set up for the different γ-glutamyl dipeptides in negative ionization mode. Spray voltage was −3.0 kV, and collision energy was set at 20 ± 10 V, with a loop time of 1.0 s for 25 events. The ion source optimal conditions were: nebulizing gas flow 3.0 L/min; heating gas flow 10.0 L/min; interface temperature 300 °C; drying gas flow 10.0 L/min; desolvation line temperature 250 °C and heat block temperature 400 °C.

Authentic chemical standards of γ-Glu-Cys and glutathione (Sigma-Aldrich Chemicals Co., MO USA) were run under identical conditions to determine experimental fragmentation patterns, and corresponding matches in a public domain mass spectral database (METLIN). *m*/*z* 128.0350, representative of the deprotonated glutathione, was designated as the diagnostic fragment of the glutamic acid residue in all dipeptides. Peak detection and area measurements used LabSolutions Insight software version 3.50SP2 (Shimadzu, Japan). Results were calculated as relative quantification (i.e. between groups within each analyte) in terms of their relative peak areas on chromatograms.

### 2.5 Kokumi taste receptor response

Aliquots of WSE, PA and GA were centrifuged briefly to clear debris. The supernatant was further centrifuged at 10,000 × *g* for 45 min and passed through < 10 kDa MWCO filter to collect ultrafiltrate containing soluble proteins and amino acids. Total protein concentration was determined with a BCA assay kit (Thermo Fisher Scientific, MA USA). The ultrafiltrates were stored at-80 °C until used for further analysis.

Mammalian cell lines, CHO-K1-CaSR (stably expressing recombinant human CaSR; #M00434, GenScript, Piscataway, NJ, USA) and CHO-K1-Gα15 as a negative control (stably expressing Gα15 protein; #M00257) were purchased from Genscript, USA and cultured in Ham’s F12, Glutamax media (#31765035, Gibco, TX, USA) supplemented with 10% FBS, penicillin-streptomycin and selection antibiotics (Zeocin (200 μg/mL) & hygromycin (100 μg/mL))respectively. The cells were grown at 37 °C, 5% CO_2_. The day before the experiment, approximately 70,000 cells (100 µL) were seeded in each well of a 96-well plate (#CLS3603, black/clear flat bottom, Corning, NY, USA).

Kokumi receptor activation was assessed by measuring intracellular calcium level through Fluorescent Imaging Plate Reader assay (FLIPR, Molecular Devices) as previously described by Kim et al. (2021). Briefly, when cells had reached 70–80% confluency, 100 µL of fluorescent calcium indicator dye (Molecular Devices, CA USA) was preloaded for 2 hr at 37 °C, and 5% CO_2_ followed by the addition of serially diluted ultrafiltrate samples. Subsequently, fluorescence was recorded using a FlexStation® 3 Microplate Reader (Molecular Devices, CA, USA).

Kokumi receptor activation was assessed by measuring intracellular calcium level through Fluorescent Imaging late Reader assay (FLIPR, Molecular Devices) as previously described by Kim et al. (2021). Briefly,when cells had reached 70–80% confluency, 100 µL of fluorescent calcium indicator dye (Molecular Devices, CA USA) was preloaded for 2 hr at 37 °C, and 5% CO_2_ followed by the addition of serially diluted ultrafiltrate samples. Subsequently, fluorescence was recorded using a FlexStation® 3 Microplate Reader (Molecular Devices, CA, USA).

### 2.6 Statistical analysis

All measurement were conducted in triplicate for each independent sample. One-way ANOVA was used to test for the difference between groups (Minitab 18, State College, PA USA). Tukey’s test was used to determine the statistical difference among the means (*p* < 0.05). Results are presented as mean ± standard error of mean (SEM) in tables and graphs. To calculate EC_50_ of different dose response curves in receptor assay, nonlinear regression analysis was performed using Graph pad prism software-version 9. The data was normalized and EC_50_ was calculated by using equation “Y = 100*(X^HillSlope)/(EC_50_^HillSlope + (X^HillSlope))” where X and Y represent axis. Subsequently, CaSR activity was presented on the scale of 0% and 100% against concentration (mg/mL) of ultrafiltrates.

## 3 Results and discussion

### 3.1 Free amino acids concentrations

**Fig. 1:**
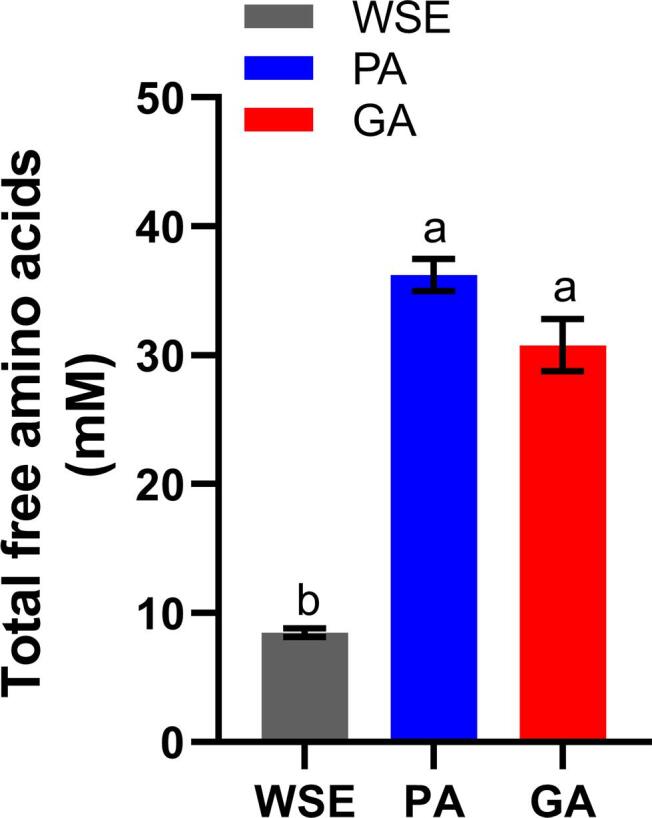
Concentrations of total free amino acids (mM) in homogenate and hydrolysates prepared from trimmed meat crusts of dry-cured lamb legs. WSE, water homogenate; PA, protein hydrolysate; GA, glutamylated samples. a,b Significant differences between the groups (p < 0.05).

**Fig. 2:**
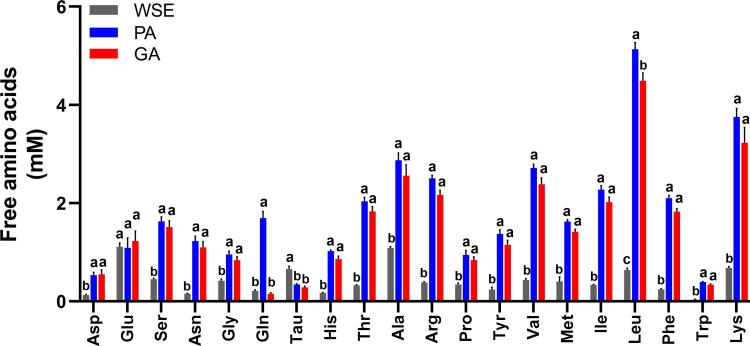
Concentrations of free amino acids (mM) in homogenate and hydrolysates prepared from trimmed meat crusts of dry-cured lamb legs. WSE, water homogenate; PA, protein hydrolysate; GA, γ -glutamylated samples. a,b,c Significant differences between WSE, PA and GA groups (p < 0.05).

FAA are not naturally abundant in meat (Triki, Herrero, Jiménez-Colmenero, & Ruiz-Capillas, 2018) but can be released by hydrolysis. The total FFA content in WSE from crusts of dry-cured lamb legs was low at 8.47 ± 0.34 mM (Fig. 1). This was markedly increased by enzyme treatments, with PA and GA significantly greater at 36.22 ± 1.25 and 30.78 ± 2.02 mM, respectively. The majority of individual amino acids were increased (Fig. 2). The most abundant were Leu, Lys and Ala.

The specificity of GGT was clearly demonstrated by changes in the concentration of Gln. Free Gln in WSE that was increased in the PA hydrolysate was fully consumed by the activity of GGT in GA. The concentration of free Glu in WSE was 5-fold greater than Gln, but concomitant changes in PA and GA were not observed. Concentrations of Gln and Glu are not expected to be reciprocal. γ-glutamyl transpeptidase catalysis of Gln includes transpeptidation, auto-transpeptidation and hydrolysis, and only the latter leads to Glu (Saini, Kashyap, Bindal, Saini, & Gupta, 2021).

### 3.2 Evaluation of γ-glutamyl peptides

Supplementary Fig. 1 shows fragmentation patterns of standards γ-Glu-Cys and glutathione run under our experimental conditions, and the corresponding fragmentation pattern reported in METLIN. Based on this data, *m*/*z* 128.0350 was designated as the diagnostic fragment of the glutamyl residue for all gamma-glutamyl dipeptides (Supplementary Table 1) in accordance with Wikoff, Kalisak, Trauger, Manchester, and Siuzdak (2009).

An exemplar of detection and quantification of the glutamyl residue in a dipeptide from a sample is shown in Supplementary Fig. 2. In this case, the glutamyl residue in the dipeptide gamma-glutamyl-alanine from a glutamylated sample (GA) was detected and quantified based on the MRM transition of *m*/*z* 216.0990 → 128.0350. The peak corresponding to the *m*/*z* 128.0350 fragment was integrated and quantified. Likewise, peaks corresponding to the diagnostic fragment in all dipeptides (Supplementary Table 1), wherever detected, were quantified and compared.

**Fig. 3:**
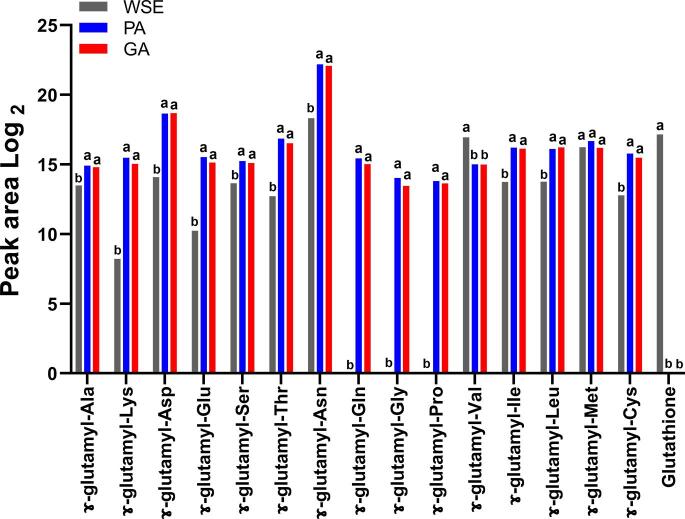
Normalised peak areas of the glutamyl residues from different dipeptides obtained from trimmed meat crusts of dry-cured lamb legs. WSE, water homogenate; PA, protein hydrolysate; GA, γ -glutamylated samples. a,b Significant differences between WSE, PA and GA groups (p < 0.05).

Fig. 3 shows the relative quantification of 15 γ-dipeptides and a glutathione (γ-Glu-Cys-Gly) that were identified in the homogenate and hydrolysates. Glutathione and γ-Glu-Val already existed in the meat crusts; their concentrations in WSE were higher than in PA and GA. Relative concentrations (Peak area Log_2_) of the remaining γ-Glu dipeptides were 1.1–4.7 folds higher in PA than in WSE. In particular, γ-Glu-Glu, γ-Glu-Gly and γ-Glu-Pro were not presented in WSE. Unexpectedly, there was no further increase in γ-Glu dipeptides in GA. It has been reported that addition of 10 and 20 mM Gln into protein hydrolysate promoted the γ-Glu dipeptides when Glutaminase SD-C100S was treated (Li, Liu, et al., 2020). It is possible that the Gln concentration at 17 mM in PA (Fig. 1) might be not enough to be support γ-Glu dipeptides formation when Glutaminase SD-C100S was treated.

γ- glutamyl dipeptides are considered as indicators of long aged dry-cured meat samples (Sforza, Galaverna, Schivazappa, Marchelli, Dossena, & Virgili, 2006). Their occurrence might be a consequence of endogenous meat enzymes or the result of microbial action during ‘fermentation’ that occurs during ageing. In this study, the degree of endogenous protein hydrolysis (reflected in the concentrations of FAA in WSE) may not have been sufficient to support the formation of γ- glutamyl dipeptides.

### 3.3 Kokumi taste receptor response

**Fig. 4:**
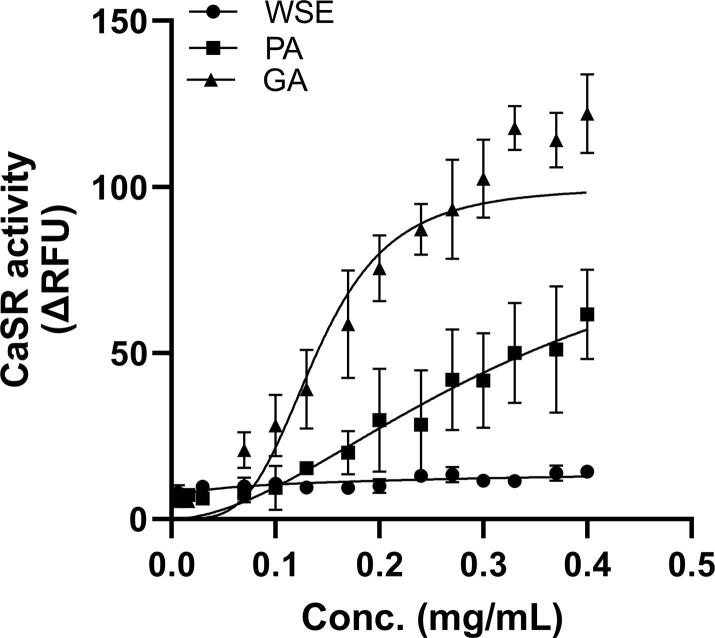
Graph shows CaSR activation response as relative fluorescent units (ΔRFU) of water-soluble extract (WSE), Protease A (PA) and glutaminase (GA) treated ultrafiltrate. Mean ± SEM, of three independent experiments. Relative EC_50_ values for GA (>0.14 mg) and PA (>0.34 mg) were calculated by non-linear regression analysis model of normalized response and variable slope.

γ-glutamyl peptides and aromatic l-amino acids bind allosterically to activate CaSR resulting in an increased intracellular calcium flux (Ahmad and Dalziel, 2020). To determine the abundance and functional taste response of kokumi di-peptides and related tastants, we compared the potency and efficacy of WSE, PA and GA to activate the CaSR. As Fig. 4 shows, WSE did not activate the CaSR even at the highest concentration, whereas the receptors responded to PA and GA in a dose-dependent manner. The GA contained higher concentration of kokumi tastants when compared with PA as shown by lower half maximal effective concentration (EC_50_) (0.14 mg versus 0.34 mg) suggesting that GGT treatment generated potent kokumi tastants in meat crust, resulting into robust CaSR activation. It is noteworthy to mention that in absence of clear upper plateau of dose response curves there is an uncertainty in EC_50_ values, and they are likely to be higher than expected (95% confidence interval for GA; 0.11 mg − 0.16 mg and PA; 0.32 mg − 0.38 mg) as discussed elsewhere (Sebaugh, 2011).

In addition to γ- glutamyl dipeptides, derivatives of amino acids and peptides can contribute to the kokumi taste, including sulphur-containing amino acids, *N*-acyl-Tyr derivatives, *N*-acetylated amino acids and Maillard reaction products (Li, Zhang, & Lametsch, 2020). We have not quantified these additional kokumi tastants in our experimental groups; whether they contributed to CaSR activation warrants further investigation. The likely presence of these different kokumi taste ligands and their varying potency to activate CaSR, could explain its stronger response to GA than PA, even though the groups did not differ in their concentrations of γ-glutamyl dipeptides. Mass spectrometry and FAA data support this notion where significant increase (p < 0.01) in GGP and kokumi relevant amino acids (Trp, Ser, His, Tyr and Phe) was observed (Fig. 2). It is also possible that γ-glutamyl polypeptides formed via the enzymatic processes were not detected by our analytical method but nevertheless stimulated CaSR. Recently we reported a positive association between CaSR activation and consumer perception of enhanced flavour in fermented meat products (Kim et al., 2021), suggesting that functional kokumi receptor activation is indicative of enhanced flavour in foods contributed by kokumi taste attributes (thickness, continuity and mouthfullness). Thus, the *in vitro* assay technique is expedient for screening and quantifying relative kokumi tastant abundance in food products. However, to validate these findings a correlation of the dose dependent receptor response of food substrate with human sensory perception studies must be conducted.

## 4 Conclusion

This is the first study demonstrating application of *in vitro* cell based CaSR assay to validate the functionality of kokumi taste di-peptides in meat crust substrate. Based on our results we confirmed that endogenous enzymes existing in meat crust seldom contributed to kokumi tastants generation during the natural aging and ripening process of dry-cured lamb, whereas the addition of protease and γ-transferase significantly enhanced FAA and γ-glutamyl-dipeptides which led to strong CaSR activation. We propose CaSR activation as a prerequisite before performing more expensive, lengthy and time taking fundamental sensory perception studies to confirm the likely abundance of kokumi peptides in food products. Further, as a next step future investigation must be focused on validation of CaSR activation by complex homogenous substrate and its correlation with kokumi taste attributes (thickness, continuity & mouthfullness) in sensory studies.

### CRediT authorship contribution statement

**Jihan Kim:** Conceptualization, Investigation, Writing – original draft, Project administration. **Raise Ahmad:** Conceptualization, Investigation, Formal analysis, Writing – original draft. **Santanu Deb-Choudhury:** Formal analysis, Writing – review & editing. **Arvind Subbaraj:** Formal analysis. **Julie E. Dalziel:** Writing – review & editing. **Scott O. Knowles:** Writing – review & editing.

## Declaration of Competing Interest

The authors declare that they have no known competing financial interests or personal relationships that could have appeared to influence the work reported in this paper.
