# Supplementary material for: Generation and identification of kokumi compounds and their validation by taste-receptor assay: An example with dry-cured lamb meat
Source: Food Chem X. 2022 Jan 19;13:100218. doi: 10.1016/j.fochx.2022.100218 (PMC9039938; doi:10.1016/j.fochx.2022.100218)
Supplement: Supplementary data 1 [file mmc1.docx]

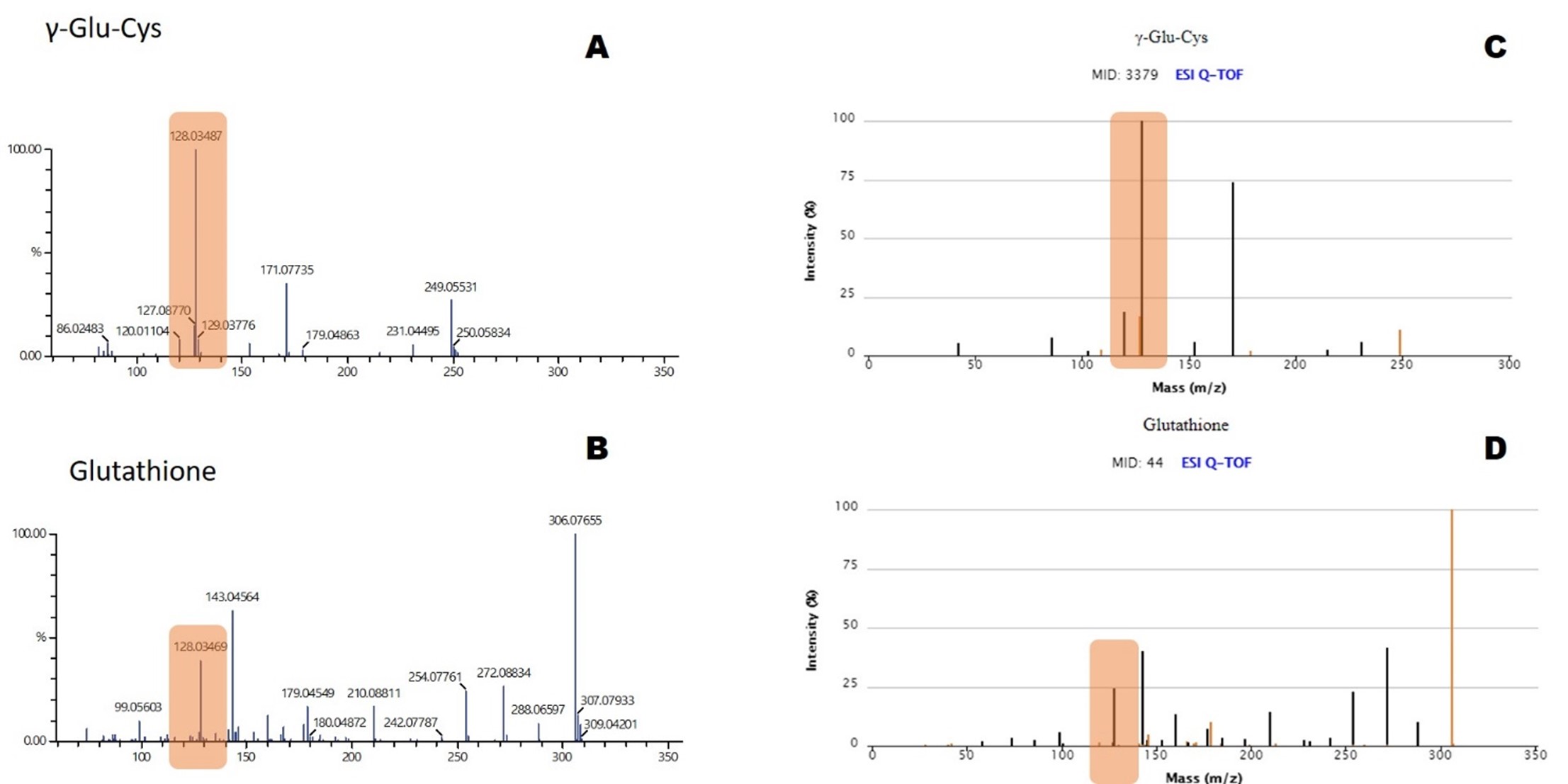


**Supplementary Figure 1.** Fragmentation patterns of authentic standards **(A)** γ-Glu-Cys and **(B)** glutathione, and the corresponding matches in METLIN database (**C and D**, respectively), showing the m/z 128.0350 fragment diagnostic of the glutamyl residue.
