# Supplementary material for: Generation and identification of kokumi compounds and their validation by taste-receptor assay: An example with dry-cured lamb meat
Source: Food Chem X. 2022 Jan 19;13:100218. doi: 10.1016/j.fochx.2022.100218 (PMC9039938; doi:10.1016/j.fochx.2022.100218)
Supplement: Supplementary data 2 [file mmc2.docx]

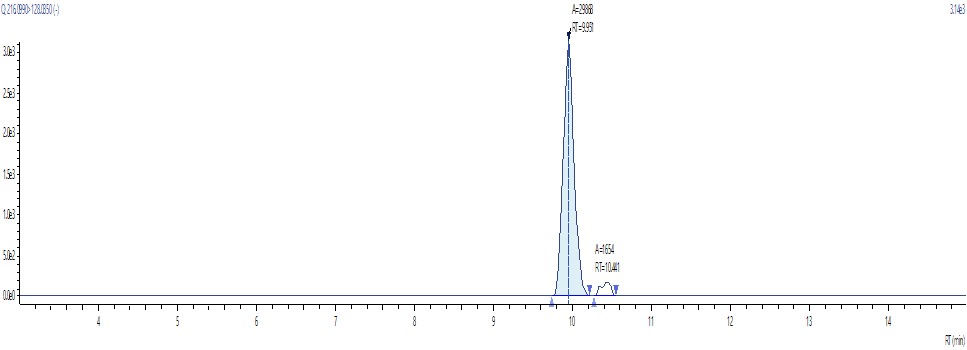


**Supplementary Figure 2.** Peak of m/z 128.0350 fragment, diagnostic of the glutamyl residue, detected and quantified from the dipeptide gamma-glutamyl-alanine in a glutamylated meat sample (G2).
