# Supplementary material for: Generation and identification of kokumi compounds and their validation by taste-receptor assay: An example with dry-cured lamb meat
Source: Food Chem X. 2022 Jan 19;13:100218. doi: 10.1016/j.fochx.2022.100218 (PMC9039938; doi:10.1016/j.fochx.2022.100218)
Supplement: Supplementary data 3 [file mmc3.docx]

**Supplementary Table 1.** MS parameters used for dipeptide analysis

| Event # | Precursor Ion m/z | Compound Name |
| --- | --- | --- |
| 1 |  |  |
| 2 | 249.0551 | Glu-Cys |
| 3 | 216.099 | Gamma-glutamyl-Alanine |
| 4 | 301.163 | Gamma-glutamyl-Arginine |
| 5 | 282.1208 | Gamma-glutamyl-Histidine |
| 6 | 273.1568 | Gamma-glutamyl-Lysine |
| 7 | 260.0888 | Gamma-glutamyl-Aspartate |
| 8 | 273.0966 | Gamma-glutamyl-Glutamate |
| 9 | 232.0939 | Gamma-glutamyl-Serine |
| 10 | 246.1095 | Gamma-glutamyl-Threonine |
| 11 | 259.1048 | Gamma-glutamyl-Asparagine |
| 12 | 273.1204 | Gamma-glutamyl-Glutamine |
| 13 | 202.0833 | Gamma-glutamyl-Glycine |
| 14 | 242.1146 | Gamma-glutamyl-Proline |
| 15 | 244.1303 | Gamma-glutamyl-Valine |
| 16 | 258.1459 | Gamma-glutamyl-Isoleucine |
| 17 | 258.1459 | Gamma-glutamyl-Leucine |
| 18 | 276.1024 | Gamma-glutamyl-Methionine |
| 19 | 292.1303 | Gamma-glutamyl-Phenylalanine |
| 20 | 308.1252 | Gamma-glutamyl-Tyrosine |
| 21 | 331.1412 | Gamma-glutamyl-Tryptophan |
| 22 | 248.0711 | Gamma-glutamyl-Cysteine |
| 23 | 306.0765 | Glutathione |
| 24 | 146.0459 | L-Glutamate |
| 25 | 128.0353 | Pyroglutamic acid |

Type = MRM(Ch1), Start minute = 3, End minute= 15, Product Ion m/z = 128.035, TOF Start m/z = 128.0324, TOF End m/z = 128.0376. Except for Event #1: Type = MS, TOF Start m/z = 55, TOF End m/z = 1100. Except for Event #25: Product Ion m/z = 82.0292, TOF Start m/z = 82.0276, TOF End m/z = 82.0308
